# DNA and RNA vaccines against tuberculosis: a scoping review of human and animal studies

**DOI:** 10.3389/fimmu.2024.1457327

**Published:** 2024-10-03

**Authors:** Alisa Kazakova, Pavel Zhelnov, Roman Sidorov, Anna Rogova, Olga Vasileva, Roman Ivanov, Vasiliy Reshetnikov, Albert Muslimov

**Affiliations:** ^1^ Translational Medicine Research Center, Sirius University of Science and Technology, Sochi, Russia; ^2^ Zheln, Toronto, ON, Canada; ^3^ Institute of Health Policy, Management and Evaluation, Dalla Lana School of Public Health, University of Toronto, Toronto, ON, Canada; ^4^ Institute of Ecology and Genetics of Microorganisms, Perm Federal Research Center, Russian Academy of Sciences, Ural Branch, Perm, Russia; ^5^ Saint-Petersburg State Chemical-Pharmaceutical University, St. Petersburg, Russia; ^6^ Laboratory of Nano- and Microencapsulation of Biologically Active Compounds, Peter The Great St. Petersburg Polytechnic University, St. Petersburg, Russia

**Keywords:** tuberculosis, DNA vaccine, RNA vaccine, BCG, adaptive immune response, scoping review, vaccine development

## Abstract

**Introduction:**

To comprehensively identify and provide an overview of *in vivo* or clinical studies of nucleic acids (NA)-based vaccines against TB we included human or animal studies of NA vaccines for the prevention or treatment of TB and excluded *in vitro* or *in silico* research, studies of microorganisms other than *M. tuberculosis*, reviews, letters, and low-yield reports.

**Methods:**

We searched PubMed, Scopus, Embase, selected Web of Science and ProQuest databases, Google Scholar, eLIBRARY.RU, PROSPERO, OSF Registries, Cochrane CENTRAL, EU Clinical Trials Register, clinicaltrials.gov, and others through WHO International Clinical Trials Registry Platform Search Portal, AVMA and CABI databases, bioRxiv, medRxiv, and others through OSF Preprint Archive Search. We searched the same sources and Google for vaccine names (GX-70) and scanned reviews for references. Data on antigenic composition, delivery systems, adjuvants, and vaccine efficacy were charted and summarized descriptively.

**Results:**

A total of 18,157 records were identified, of which 968 were assessed for eligibility. No clinical studies were identified. 365 reports of 345 animal studies were included in the review. 342 (99.1%) studies involved DNA vaccines, and the remaining three focused on mRNA vaccines. 285 (82.6%) studies used single-antigen vaccines, while 48 (13.9%) used multiple antigens or combinations with adjuvants. Only 12 (3.5%) studies involved multiepitope vaccines. The most frequently used antigens were immunodominant secretory antigens (Ag85A, Ag85B, ESAT6), heat shock proteins, and cell wall proteins. Most studies delivered naked plasmid DNA intramuscularly without additional adjuvants. Only 4 of 17 studies comparing NA vaccines to BCG after *M. tuberculosis* challenge demonstrated superior protection in terms of bacterial load reduction. Some vaccine variants showed better efficacy compared to BCG.

**Systematic review registration:**

https://osf.io/, identifier F7P9G.

## 1 Introduction

Every year, ~10 million people become ill with tuberculosis (TB) and 1.5 million die. These data make TB the leading infectious cause of death in the world, despite being a preventable and treatable disease (1). Unfortunately, the treatment of TB is complicated by the growing antibiotic resistance of *Mycobacterium tuberculosis* (*Mtb*), which is the causative agent of TB (1). At the moment, the WHO TB Strategy is in effect, stipulating a 95% reduction in TB mortality and a 90% reduction in TB incidence worldwide by 2035. To achieve these goals, a new vaccine effective for all age groups, especially for adults and adolescents, will be required. Vaccines also offer the best chance of stopping the accelerating spread of multidrug-resistant TB (1).

The first step for the development of anti-TB vaccines was made in 1882, when Robert Koch described the TB etiological agent (2), which has served as a platform for the creation of vaccines, diagnostics, and therapies. Furthermore, Koch in 1890 attempted to vaccinate against TB for the first time and developed so-called “tuberculin” (3). The latter did not show effectiveness as either a therapy or as a vaccine against TB but turned out to be an excellent diagnostic tool. It was a subunit vaccine consisting of protein antigens and various glycolipids that was administered subcutaneously, and those infected with TB had a characteristic reaction after 2–3 days (delayed hypersensitivity reaction) (4). For the modern diagnosis of TB, an improved version of tuberculin is used: a tuberculin skin test (TST) based on a purified protein derivative (PPD).

After many attempts to create a vaccine against TB, Calmette and Guérin concluded that subunit vaccines and killed whole-cell vaccines would not provide sufficient protection because the attenuation of the pathogen was unstable. The first and only effective anti-TB vaccine was the *Bacillus* Calmette–Guérin vaccine (BCG), originally developed at the Pasteur Institute in France in 1921 to reduce infant mortality from TB and was used in newborns. BCG is a live attenuated strain of *Mycobacterium bovis* obtained by continuous cultivation under debilitating conditions (5). Previous attempts to create a vaccine for tuberculosis, such as thermal or chemical inactivation of the tuberculosis bacillus, have proven ineffective. It appears necessary to use a live vaccine. Work conducted by Nobel laureate Emil von Behring in 1902 showed that inoculation with human tuberculosis strains could protect cattle from bovine tuberculosis. However, subsequent research revealed that potentially infectious, viable bacilli were excreted in milk (5). Based on this knowledge, Calmette and Guérin began searching for a vaccine for humans. While trying to cultivate the tubercle bacillus for experimental use, they noticed that using a standard potato-glycerin nutrient medium led to an undesirable accumulation of bacteria. They tried adding bovine bile as a solution and, by happy coincidence, found that it not only reduced the formation of clumps but also decreased virulence during subsequent cultivation (6). In 1908, starting with the *Mycobacterium bovis* strain, the causative agent of bovine tuberculosis, researchers began a cultivation process that led to the creation of BCG. They used potato medium, glycerin, and bovine bile to create new subcultures every three weeks. This process, known as passivation, continued for 30 passages before a strain was created that was no longer lethal to guinea pigs. In 1913, plans were made to conduct a vaccination trial on cattle, but these were interrupted by the outbreak of the First World War. Despite difficulties in obtaining potatoes and bovine bile due to the German occupation of Lille, the researchers managed to preserve their research. By 1919, the “bile rod” had been passed through 230 generations without causing tuberculosis in rabbits, guinea pigs, or cattle. At this point, Calmette and Guérin believed that the bacteria were sufficiently weakened not to cause disease in humans, but instead could stimulate an immune response that would provide immunity against tuberculosis. The opportunity to conduct the first human trial presented itself in 1921 thanks to Doctors Weil-Halle, H. Morrison, and H. McShane, who worked at the Charité Hospital in Paris. They contacted Calmette about a healthy newborn whose mother had died of tuberculosis soon after giving birth. On July 18, 1921, the infant became the first person to receive a dose of BCG (5). Calmette believed that the natural route of *Mycobacterium tuberculosis* infection was through the gastrointestinal tract, so BCG was administered orally at first. No adverse effects were observed, and the child lived without tuberculosis (7).

Even a century after the development of BCG, it is still the only licensed vaccine for the prevention of TB and successfully precludes severe forms of TB in children. The most recent data indicate that 153 countries have a policy of BCG vaccination for the whole population: 87 of these countries have reported coverage of at least 90% of residents (1), http://www.bcgatlas.org/. Unfortunately, BCG has multiple drawbacks, which greatly affect its protective properties. First of all, its effectiveness is estimated at 0% to 80% in different geographic regions (8), and in tropical countries, the protection is much lower than at higher latitudes. Additionally, there are many other factors that determine the immunogenicity of BCG: differences in the effectiveness of the vaccine may be caused by residence in rural or urban areas (9), the sex of the vaccinated (10), the risk of TB in a study population (11), the content of mycobacteria in the environment (12), and other determinants. Another disadvantage of BCG is its heterogeneity: each of the 22 strains used for vaccination (13) varies within a substrain obtained from different sources (14), and these dissimilarities lead to variability of immunogenicity (6). In addition, BCG lacks several immunodominant antigens, such as ESAT6, CFP10, and the type VII ESX-1 system, because of the loss of the RD1 region (15–17). Moreover, in spite of BCG-mediated protection against miliary and meningeal forms of TB in children, the effectiveness of BCG decreases 20 years after vaccination (18, 19), and BCG does not provide protection from pulmonary TB in adults (20, 21). Although BCG revaccination has an efficacy of 45.4% in the prevention of *Mtb* infection (22), it is not recommended by the WHO for use. Another disadvantage is that the effectiveness of BCG is affected by insufficient induction of CD8^+^ T cells (23), which play an important role in the protection against TB, as well as CD4+ T cells. Furthermore, BCG vaccination is not applicable to people with HIV because the risk of BCG disease in HIV-infected people has increased several-hundred-fold (24). Finally, there is no strong evidence that BCG is effective as a therapeutic vaccine.

Despite all the disadvantages, BCG has remained one of the most cost-effective means of TB prevention in countries with high incidence of TB for a long time (25), because so far, no new anti-TB vaccine has been licensed. Currently, there is no vaccine that would be effective at the prevention or treatment of TB in adults (24); these properties may affect the number of TB cases in the future.

The new class of therapeutic vaccines are nucleic acid (NA)-based vaccines (hereafter: NA vaccines), which consist of genes encoding *Mtb* antigens. The specific feature of NA vaccines compared to BCG is that they do not contain a potentially infectious agent, and therefore they can take advantage of antigens sourced directly from a target species. Vaccines should be specific to a certain pathogen, and thus available DNA vaccines are mostly based on *Mtb* antigens, but there is an example of a DNA vaccine expressing the *Mycobacterium leprae* HSP65 protein to provide immunity against *Mtb* in a mouse model (26).

The purpose of our review was to perform the first systematic examination of the literature in the area of NA synthetic TB vaccines. Moreover, here we present a discussion of approaches that can improve the effectiveness, immunogenicity, and stability of an RNA vaccine against *Mtb*.

The use of systematic methodology has become a standard approach to synthesizing evidence about health interventions, e.g., preventive vaccines for COVID-19 (27). The applicable systematic methodology varies by purpose of synthesis (28). We employed a free Web-based tool (29) to choose the most appropriate type of evidence synthesis for the purpose of this review, and the tool suggested a rapid scoping review (30, 31). Nonetheless, after carefully weighing ref (32). against our timeframe and resources and because of the success of the recent “2-week review” initiative (33), we opted for conducting a full scoping review via a modified 2weekSR approach.

There is a striking difference in how the systematic methodology is prevalent in the syntheses of clinical evidence but not within reviews of preclinical research. With some focus and recent initiatives pertaining to systematic reviews of animal studies (34, 35), few examples of other types of evidence synthesis or systematically synthesized *in vitro* research (36) can be found in the literature. As a rough measure of prevalence as of March 25, 2024, PubMed returned only 5 records mentioning “tuberculosis,” “vaccines,” and “scoping” (“tuberculosis”[tw] AND “vaccine*”[tw] AND “scoping”[tw]), and 19 records mentioning both “mRNA” and “scoping” (“mrna”[tw] AND “scoping”[tw]), with none of these being relevant to the target subject of the search; therefore, this study appears to be warranted.

## 2 Materials and methods

### 2.1 Review design

A scoping review was conducted from a protocol prospectively registered on March 31, 2022 (37). The “Right Review” tool was used to inform the review design (29). The conduct was guided by The JBI Manual for Evidence Synthesis (38) and a modified 2weekSR approach (39).

### 2.2 Eligibility criteria

The populations of interest are patients with TB and animal models of TB. We excluded studies of bacteria other than *M. tuberculosis*, such as *M. bovis*, *M. leprae*, or agents of mycobacterial diseases of humans and animals, as well as any applications outside of TB prevention or treatment, e.g., in oncology or veterinary medicine. Therapeutic TB vaccines were a post-protocol addition because we did not initially anticipate them to be a sizeable field of study. Any vaccines based on NA (mRNA, DNA, or other) were eligible. Studies of vaccines based on viruses, such as modified vaccinia virus Ankara (MVA)-based vaccines, were excluded. Any *in vivo* or clinical studies were eligible, but we abandoned our initial intention to include *in vitro* and *in silico* studies to focus on the many animal studies we identified. We imposed no limitations on the publication date. Any publication types were eligible for inclusion. We excluded reports that did not provide original primary research data or were insufficient to complete our predefined data chart, such as reviews, letters, or low-yield conference abstracts. We translated any reports in languages other than English using DeepL or Google Translate (Lens).

### 2.3 Search

We conducted initial searches in databases and registers on May 1-3, 2022. These included academic databases such as PubMed and Embase, preprint databases, animal or veterinary study databases, and clinical trial registers. At the protocol stage, we reviewed the websites of the Tuberculosis Vaccine Initiative, the Virtual Global Forum on TB Vaccines 2021, and the 6^th^ Global Forum on TB Vaccines 2022 for any additional sources of evidence but identified none. All searches were conducted in English only. A comprehensive listing of the sources of evidence searched, as well as the associated search strategies, is presented in the protocol (37), and the list of post-protocol changes is available from the review repository. Our search strategy for PubMed underwent PRESS peer review (40) by an information specialist, who declined public acknowledgment, and was revised accordingly before the protocol publication. We conducted additional searches across the same databases and registers and in Google on September 21, 2022, using the list of vaccine names compiled after record screening. We manually scanned full texts of literature reviews identified during screening for relevant references but canceled forward citation tracking. We did not contact study authors.

### 2.4 Study selection

Duplicate records were removed using Deduplicator (41), Rayyan (42), and manually. Two reviewers screened each record independently and in duplicate, using Rayyan if possible or by hand otherwise. Before screening, several team members assessed 20 randomly selected records to develop a shared understanding of how to apply the eligibility criteria. Further, we randomly selected another 200 records and conducted piloting for all pairs of screeners. Screening was conducted in two stages: by title and abstract (if present) and by full text. Disputes were resolved by discussion.

### 2.5 Data charting

PDF copies of reports stored in Rayyan were charted using a custom installation of FormTools (https://formtools.org/). Due to a large number of included reports, data charting was not conducted in duplicate as originally planned. A.K., R.S., V.R., and A.M. completed principal charting, A.M. and V.R. cross-checked charts for factual errors, and P.Z. validated the data integrity. All form fields were optional to fill, and the number of sessions was not limited. A copy of the data charting form is available from the review repository.

### 2.6 Synthesis

We descriptively summarized the content of the data charts and grouped it in a manner naturally emerging from the chart content and richness. No critical appraisal was performed because evidence on vaccine efficacy was not formally synthesized, which aligns with the JBI Manual guidance for scoping reviews (38). Bibliographic information was collected from OpenAlex (43), and visualizations were done in Microsoft Excel.

### 2.7 Reporting

The review is reported in accordance with PRISMA-ScR (44), PRISMA-S (the filled-in checklist is reported in **Supplementary Table 2**) (45), and a preliminary version of the PRISMA Extension for Preclinical *In Vivo* Animal Experiments checklist (the filled-in checklist is reported in **Supplementary Table 1**) (34). We consulted PRISMA 2020 for Abstracts (46) and Claude Free plan (https://claude.ai/ accessed on June 14, 2024) while composing the abstract. The flow diagram was generated with the PRISMA2020 R package (47). CRediTas 0.2.0.9 was used to generate the authorship statement https://docs.ropensci.org/CRediTas. An extended version of the methods report is available from the review repository. A video report is available from the Write In Stone research transparency system (https://my.writeinstone.com/public/research/published-3b616e83-a764-4c18-92fe-88a465604d0e).

### 2.8 Review updates

We do not plan to update this review but are open to collaboration with researchers willing to do so.

## 3 Results

### 3.1 Study selection and characteristics

**Figure 1:**
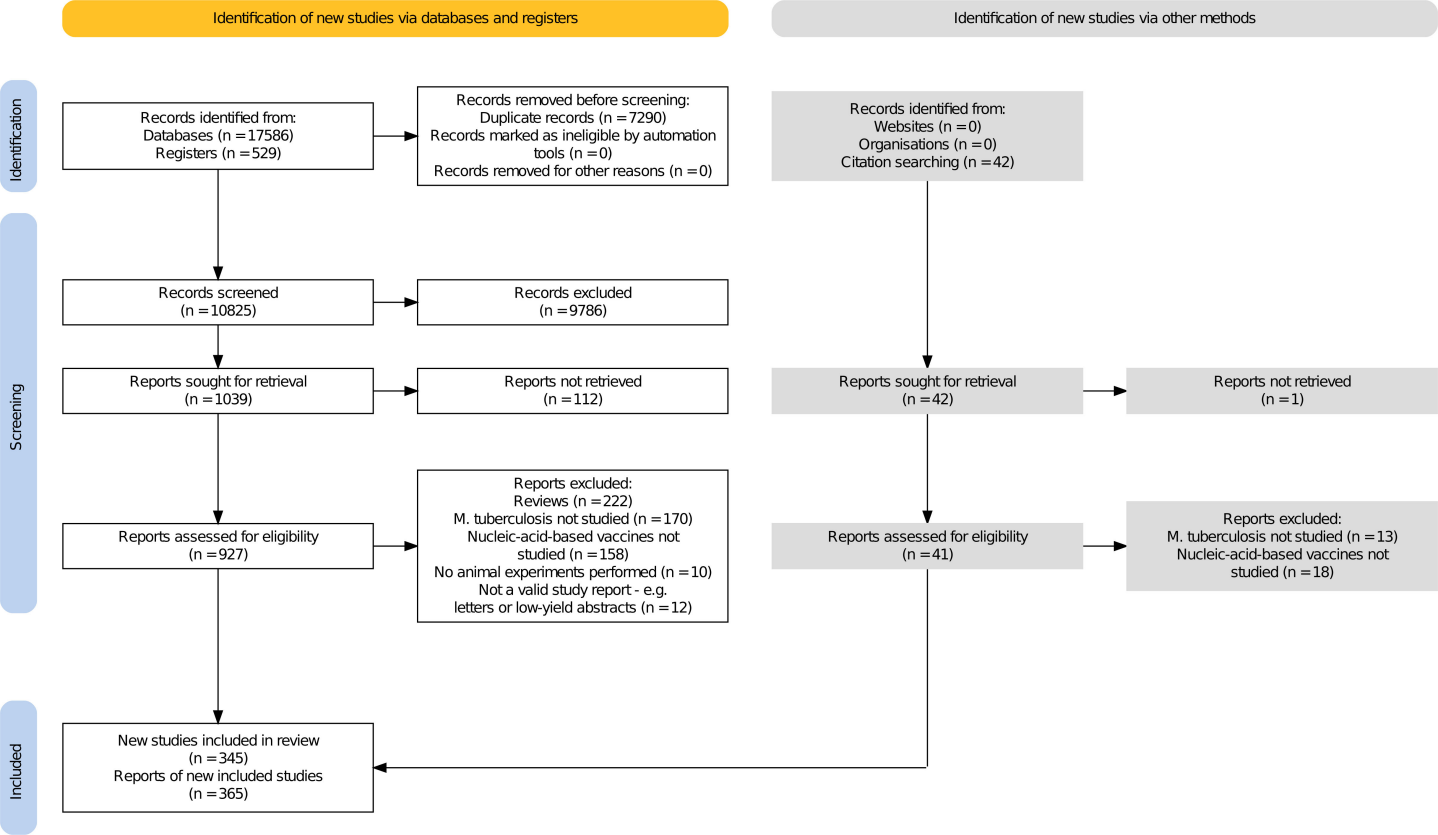
PRISMA 2020 flow diagram.

The study selection process is summarized in a flow diagram (**Figure 1**).

A total of 1081 records were included and sought for full-text retrieval. We failed to retrieve reports for 113 of them, mostly due to our restricted access to Chinese academic databases. Of the 968 reports retrieved, 39 were in languages other than English, all of which were successfully auto-translated. 17 of these were in Chinese, 9 in Japanese, 4 in French, and 3 in Spanish. 6 reports were in a different language each (German, Korean, Persian, Portuguese, Russian, or Turkish). Following screening, 365 reports of 345 studies were included in the review. The reasons for exclusion were noted by reviewers as free-text Rayyan labels and then collated by P.Z. in a hierarchical order: reviews (222 reports), studies of microorganisms other than M. tuberculosis (170 reports), irrelevant types of vaccines (158 reports), relevant but not reporting any animal studies (10 reports), and low-yield abstracts or commentaries (12 reports).

**Figure 2:**
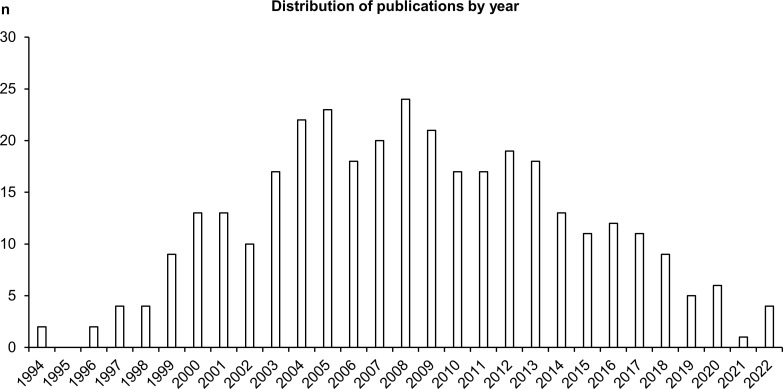
Distribution of the included studies by publication year.

Among the included studies, most were published between 2001 to 2015 (first and third quartiles; median: 2008) (**Figure 2**). The top three first-author country affiliations, exclusive of unknown and multiple values, were China, the United States, and Brazil.

### 3.2 Antigens

**Figure 3:**
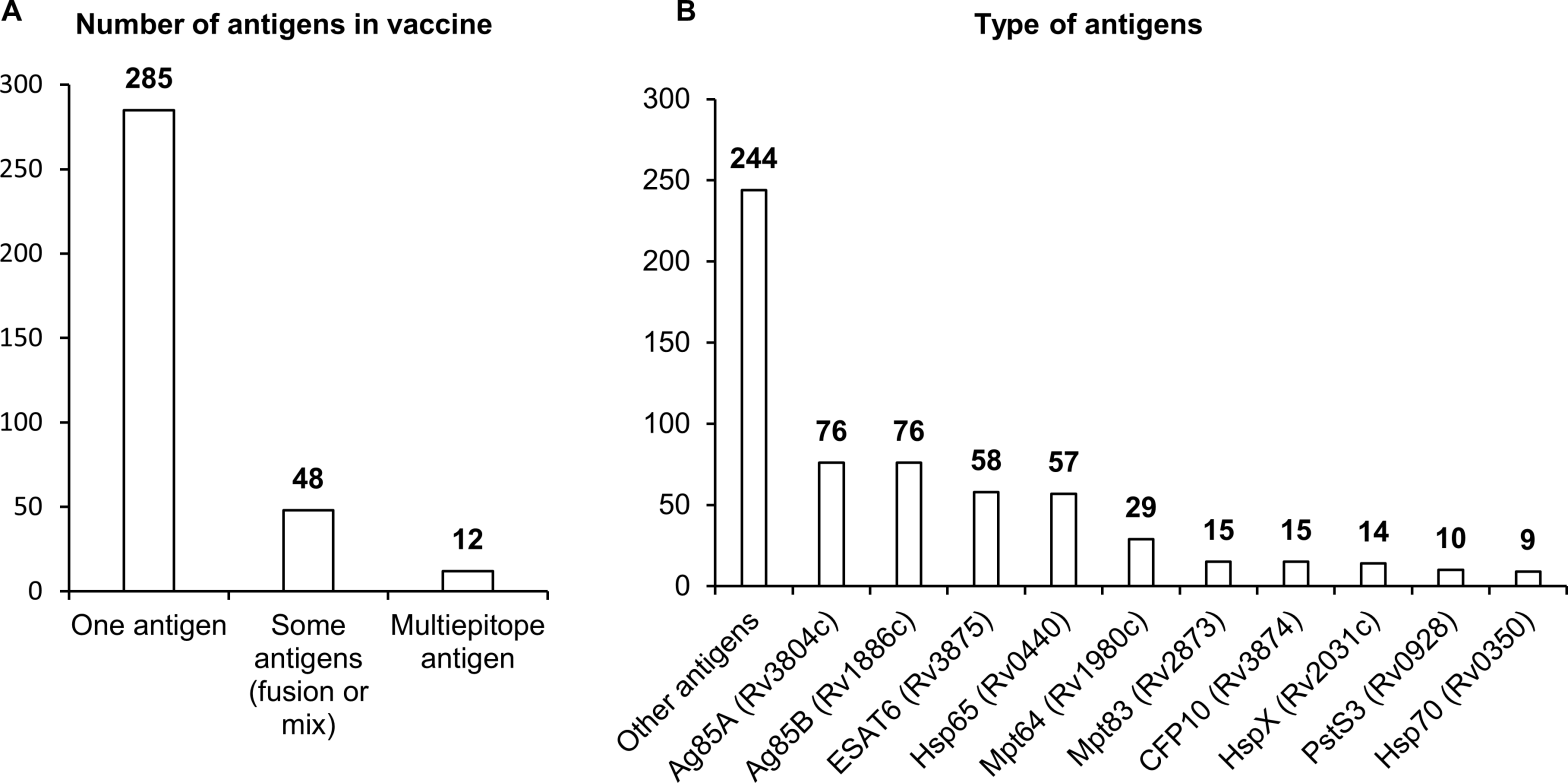
Antigens used in NA vaccines against TB. **(A)** The number of antigens in a vaccine. **(B)** Types of antigens. The numbers indicate the number of studies in which these antigens have been used.

Of the 345 studies included in the final analysis, the vast majority (285) have involved single-antigen vaccines. In 48 studies, researchers have used two or more different antigens or a combination of an antigen and an encoded adjuvant including sequences of genes *IL12*, *CD226*, *GMCSF*, *IL33*, and *IL21*. Only 12 studies have involved multiepitope vaccines (**Figure 3**).

There are three distinct groups of antigens that have been most frequently used in vaccines against *Mtb*. The first group contains sequences coding for immunodominant secretory antigens Ag85A, Ag85B, ESAT6, MPT64, and CFP10. Researchers have most often employed sequences of genes *Rv3804c* and *Rv1886c*, which encode secreted fibronectin-binding proteins Ag85A and Ag85B possessing mycolyltransferase activity necessary to maintain the integrity of the mycobacterial cell wall. The Ag85A protein is conserved across mycobacterial species including *Mtb*, BCG, and environmental mycobacteria, and this situation allows to apply vaccines based on them against most of virulent strains of mycobacteria. Sequences of genes *Rv3875*, *Rv1980c*, and *Rv3874* coding for highly immunogenic secreted proteins ESAT6, CFP10, and MPT64 have also been used in many studies. *ESAT6* and *CFP10* are a part of the RD1 region of difference between *Mtb* and BCG, are absent in strains used for BCG, and are widely used in the diagnosis of TB in the clinic (48–50). *Mpt64* belongs to the RD2 region of difference and is present in some BCG strains (51).

The second group of most popular sequences in DNA or RNA vaccines against *Mtb* includes sequences of genes *Rv0440*, *Rv0350*, and *Rv2031c*, which encode heat shock proteins (HSPs) HSP65, HspX, and HSP70. HSPs are ubiquitous chaperones that are induced by heat, pH extremes, oxygen or nutrient deprivation, and other environmental stressors. HSPs maintain long-term survival of mycobacteria; besides, in response to stress or after cell death, HSPs can be actively secreted into the extracellular environment and activate innate immune responses through different cellular receptors (52, 53). HSP65 signals through receptor CD14, and HSP70 through TLR2 and TLR4, thereby stimulating the production (by monocytes) of such proinflammatory cytokines as TNF and IL-6.

The third group of sequences most often employed in vaccines includes genes *Rv2873* and *Rv0928*, coding for cell wall proteins: lipoproteins MPT83 and PSTS3. MPT83 is a surface-exposed glycosylated lipoprotein that induces strong T-cell responses and antibody generation (54). PSTS3 is a component of a putative phosphate transporter and induces the highest levels of cytokine secretion (54).

### 3.3 Delivery systems

**Figure 4:**
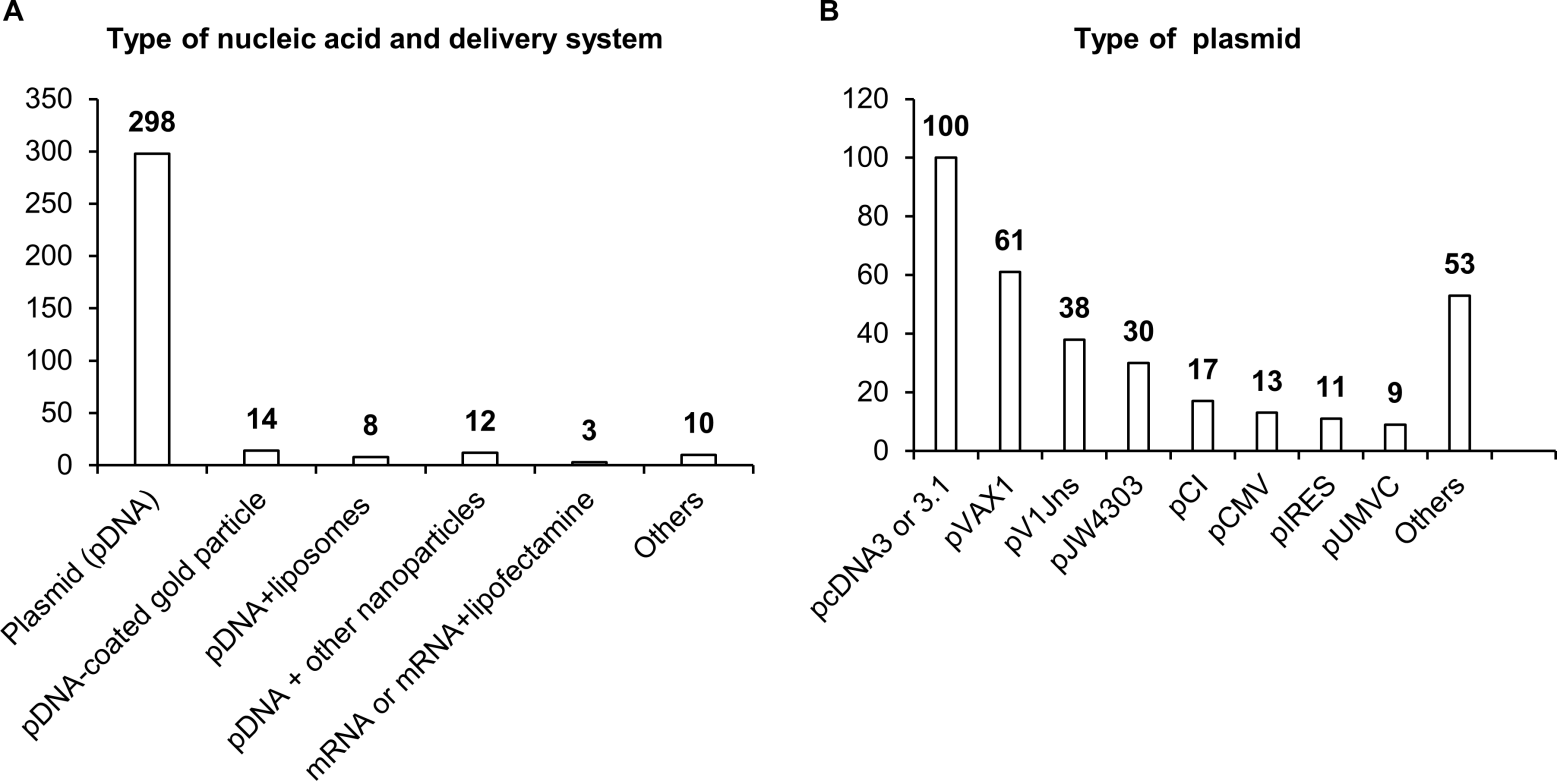
Delivery systems used in NA vaccines against TB. **(A)** Types of NA and delivery system. **(B)** Types of antigens. The numbers indicate the number of studies in which these delivery systems have been used.

Most studies have involved DNA vaccines (342 studies), and only three studies have dealt with mRNA vaccines. In the overwhelming majority of studies, naked plasmid DNA has been employed; only in 34 studies were nanoparticles, liposomes, or microspheres used to increase the efficiency of DNA delivery. As a plasmid backbone, a standard set of plasmids used in biotechnology has been utilized (**Figure 4**). Although DNA molecules, unlike RNA, are stabler and less susceptible to degradation by endonucleases, research indicates that the use of naked plasmid DNA is very ineffective, and when such DNA is administered intramuscularly, more than 95% of plasmid DNA remains in the interfibrillar space and does not enter the cells (55, 56). A nanoparticle-based NA delivery system can encapsulate negatively charged NAs, protect them from degradation by endogenous enzymes, and facilitate cellular uptake and an intracellular release (57, 58). Some nanoparticles may also have enhanced affinity for antigen-presenting cells (APCs) (57, 58).

Delivery systems based on natural polymer (d,l-lactic-co-glycolic acid) (PLGA) were used in a study by Dalirfardouei et al. (59). PLGA-based nanoparticles act as a transfection mediator, enhance the internalization by macrophages and dendritic cells (DCs), and protect plasmid DNA from nucleolytic degradation (60, 61). Polyhydroxy biopolyester nanoparticles, Fe_3_O_4_-Glu-polyethylenimine, or gold nanoparticles induce a stronger immune response, improve recognition and presentation of antigens, and have also been used in DNA vaccines against TB (62–64). After entering the cell, PLGA and Fe_3_O_4_-Glu-polyethylenimine nanoparticles slowly degrade, thereby ensuring long-acting cellular and humoral immune responses (58, 65).

A separate field of research and development of NA vaccine delivery is lipid nanoparticles. Cationic liposomes interact with a negatively charged NA and surfaces of DCs, thus ensuring effective delivery of the vaccine into the cell (66). A distinct feature of the use of lipid nanoparticles is the possibility of their “targeting” via modifications of lipid composition and/or inclusion of various protein macromolecules and antibodies into such nanoparticles (67, 68). Classic four-component composition of lipid nanoparticles (an ionizable lipid, helper lipid, cholesterol, and polyethylene glycol 2000) helps lipid nanoparticles to penetrate most organs and tissues with predominant localization in the liver (69).

Moreover, liposomes can be combined with viral vectors, for instance, with hemagglutinating virus of Japan (HVJ; Sendai virus), thereby enhancing DNA vaccine immunogenicity against TB (70) and increasing transfection efficiency—10-fold as compared to the application of liposomes or naked plasmid DNA—via the HVJ–cell fusion mechanism (70, 71). Thus, the use of relevant delivery systems in TB vaccines may significantly improve their effectiveness.

### 3.4 Adjuvants

**Figure 5:**
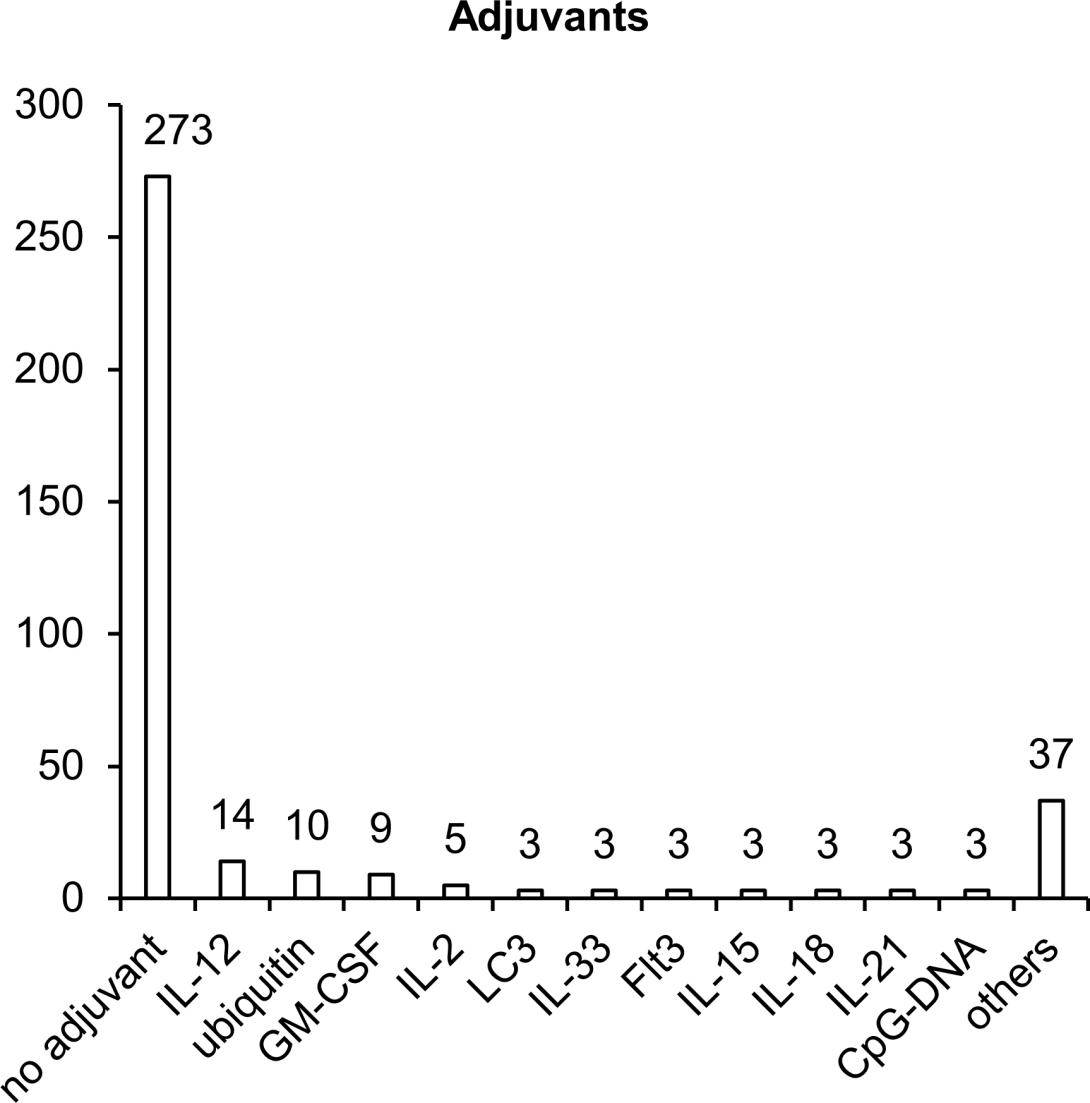
Molecular adjuvants used in NA vaccines against TB. The numbers indicate the number of studies in which adjuvants have been used.

Once inside the cell, plasmid DNA is recognized by various endosomal DNA sensors, such as Toll-like receptor 9 (TLR9) and multiple cytosolic DNA sensors DDX41, RNA pol III, DNA-PK, MRE11, cGAS, IFI16, AIM2, and DAI (72). TLR9 specifically recognizes unmethylated dinucleotide motifs CpG and activates innate immune responses through the STING–TBK1 signaling cascade (73, 74). Most of the TB vaccine development studies that we reviewed did not involve additional adjuvants (**Figure 5**). Nonetheless, to enhance immunogenic properties of NA vaccines, 21% of studies (72 articles) involved various adjuvants, which can create a microenvironment necessary for the formation of innate and adaptive immunity. Sequences encoding adjuvants have typically been included in plasmids used for vaccination. The most popular adjuvants are cytokines, chemokines, and other immunostimulatory molecules such as CpG island sequences.

The sequences most popular as molecular adjuvants encode cytokines IL-2, IL-12, IL-15, and GM-CSF, which specifically induce the proliferation of NK cells, B cells and T cells and stimulate the maturation of DCs and the production of interferon-γ (IFN-γ) (75–77). IL-2 plays a key part in the control over the differentiation of CD4^+^ T helper lymphocytes, enhances the activity of NK cells and of CD8^+^ cytotoxic T lymphocytes (CTLs), stimulates the proliferation of T cells, and enhances the production and secretion of IFN-γ (78). GM-CSF plays a critical role in the activation and maturation of DCs (79). IL-12 stimulates the secretion of IFN-γ by NK cells and by CD8^+^ and CD4^+^ T cells (80). IL-18 is a proinflammatory cytokine that activates adaptive cellular (T helper 1 [Th1], Th17, or Th2) and humoral responses (81). IL-21, IL-15, and IL-33 are cytokines with broad pleiotropic effects, can act as immunomodulators (depending on the expression of other proinflammatory and anti-inflammatory factors), and stimulate the activation of Th1/Th2 cellular and humoral immunity (82–84). Additionally, as a molecular adjuvant, investigators have used the sequence of membrane tyrosine kinase of type III (Flt3), which is a receptor for several cytokines and participates in the expansion and maturation of DCs (85). Flt3L increases the total number of leucocytes and mobilizes progenitor cells into peripheral blood (86).

Two other adjuvants (microtubule-associated protein 1-light chain 3 [LC3] and ubiquitin) enhance proteasomal and lysosomal degradation of target polypeptides to facilitate their presentation by MHC I and MHC II complexes (87, 88). Addition of CpG motifs to a plasmid results in the activation of TLR9, which—through the STING–TBK1 signaling cascade—activates B cells, promotes plasmacytoid-DC and monocyte maturation, and promotes the production of proinflammatory cytokines such as IL-12 (78, 89).

Despite the high effectiveness of molecular adjuvants in NA vaccines (90, 91), the use of such adjuvants for the development of vaccines against TB has not yet become widespread. Thus, the application of molecular adjuvants and their various combinations to vaccines against TB remains a potentially promising area for improving the effectiveness of these vaccines.

### 3.5 Methods for NA vaccine delivery

**Figure 6:**
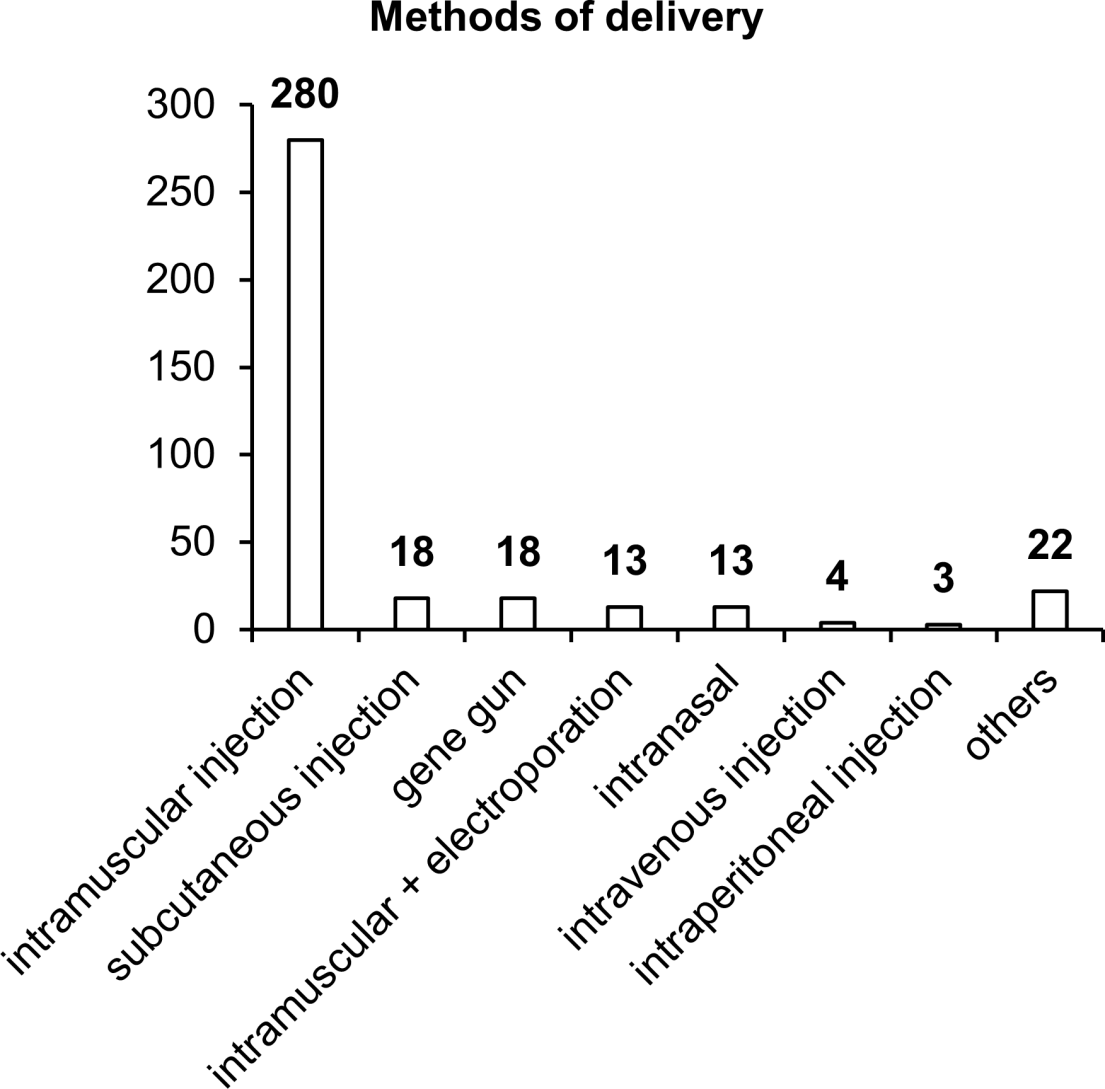
Methods of vaccine delivery. The numbers indicate the number of studies in which various delivery methods have been used.

Our results indicated that in more than 80% of the studies, intramuscular vaccine delivery has been employed, in the vast majority of cases by means of a needle and syringe, and only 18 studies have involved intramuscular delivery by electroporation (**Figure 6**). Other delivery methods such as subcutaneous injection, intranasal administration, or the gene gun have been used in only 4–5% of the studies. It is worth noting that each delivery method has its own advantages and disadvantages, and effectiveness largely depends on the delivery system utilized in the vaccine.

Intramuscular delivery of naked DNA, which has been used in most of the studies, is extremely ineffective because more than 90% of plasmid DNA does not enter cells but remains in the intercellular space. In addition, a substantial percentage of DNA gets transfected into myocytes, and only a small proportion reaches target APCs. When administered subcutaneously, plasmid DNA gets transfected into keratinocytes, fibroblasts, and various APCs, whose concentration (% of all cells) is considerably higher than that in muscle tissue (74). The intranasal delivery technique also has many advantages over intramuscular and subcutaneous administration routes (92). In the nasal cavity, the first contact with most pathogens takes place; this site is enriched with lymphatic tissue, which contains cells of systemic and mucosal immunity (92). In the nasal cavity, effective transfection of target APCs with DNA vaccines against TB is capable of inducing an appreciable immune response (93).

Improvements in such delivery methods as intramuscular and intradermal electroporation or the gene gun increase the proportion of plasmid DNA entering the cell and improve the effectiveness of vaccines (94–96). Thus, further improvement of the effectiveness of NA vaccines against TB can be achieved via modern delivery methods in combination with delivery systems.

### 3.6 The prime/boost concept and the use of synthetic NA vaccines

Our analysis of 345 studies showed that only 82 studies have dealt with homologous prime/boost vaccination or single-dose administration of an NA vaccine. In most studies, heterologous prime/boost vaccination has been utilized. Heterologous vaccination is preferable because it shows greater effectiveness and immunogenicity, especially in the context of TB vaccines: for them, an extremely important feature is their ability to stimulate a cellular immune response because it is most effective against *Mtb*. Homologous boosting usually leads to greater humoral, but not cellular, responses to target antigens, whereas heterologous prime/boost immunization affords increased efficiency of both humoral and cellular immune responses (97).

The most commonly used scheme is heterologous immunization with a viral vector or DNA vaccine for priming and with a protein vaccine as a boost; this is because the protein component causes a humoral immune response, and DNA is an inducer of both cellular and humoral responses (98, 99), thereby causing activation of both branches of an immune response. Nonetheless, there are other approaches too. For example, such regimens as DNA prime/viral vector boost, protein prime/viral boost, and viral prime/protein boost promote the induction of T-cell immune responses. VLP prime/live-vector boost allows to enhance a CD8^+^ T-cell response (100). DNA prime/VLP boost is capable of eliciting both cellular and humoral responses (101).

For an anti-TB prime/boost vaccine, researchers can employ either combinations of BCG with various synthetic vaccines or BCG-free combinations with synthetic vaccines as a prime/boost [DNA prime/protein boost (102), DNA prime/viral vector boost (103), or viral vector prime/DNA boost (104)]. Most often, regimens involving BCG prime are considered because BCG in many countries is administered in the first hours after birth. For regimens based on BCG, the antigen of the injected boost must be present in all strains of BCG and be highly conserved among all mycobacterial species; however, there are effective boosts containing the ESAT6 antigen absent in BCG. Their immunogenicity is due to the fact that ESAT6 is administered together with immunodominant antigen Ag85B as a fusion protein (105).

Prime/boost immunization against TB using BCG and synthetic vaccines can serve as preventive and therapeutic vaccination (106). A therapeutic vaccine has certain requirements: it must induce Th1-type cytokines such as IFN-γ and TNF, promote the activation of infected macrophages, and prevent reactivation of latent infection and transmission of pathogens to other people (107, 108). That is why, to create such a vaccine, it is necessary to choose early-expressed and latent-infection-associated antigens (109). Anti-dormancy antigens such as RpfB can also be used to control reactivated *Mtb* (110). Preventive vaccines against TB carry early-expressed antigens and factors triggering a T-cell immune response (111–113).

The mechanisms of the immune response that occurs after administration of the boost depend on the synthetic vaccine that is administered. Protein boosts promote the development of a pronounced antibody response, and when supplemented with adjuvants, they can induce both CD4^+^ T cells that produce INF-γ and CD8^+^ T cells, thereby inducing a cellular response (114). BCG prime/DNA boost results in the activation of CD4^+^ T cells, elevated concentrations of IFN-γ, TNF, and IL-2, an increase in the number of CD4^+^ and CD8^+^ populations, and a greater level of IgG and IgG2a antibodies at a ratio indicating a shift toward a Th1 immune response (115–117).

Thus, a boost is considered the most optimal use of DNA and RNA vaccines. Nevertheless, there is still no single most effective protocol for their use. The effectiveness of heterologous prime/boost anti-TB vaccination is influenced by other factors too (aside from the nature of the components): the order of administration of the components, the time interval between injections, the method of administration, and the presence of adjuvants in the injection mixture. The intervals between priming and boosting are important for the induction, maturation, expansion, and enhanced functioning of long-lived memory T cells (118). The route of administration is important because during infection, mucosal tissues are the first to encounter *Mtb*, and parenteral delivery of immunogens often does not elicit mucosal immune responses. It has been shown that intranasal booster vaccination of mice with fusion protein Ag85B–ESAT-6 along with adjuvant LTK63 causes a strong Th1 response and prevents the spread of infection (119). Furthermore, to increase the immunogenicity of an anti-TB prime/boost vaccine, the following approaches are employed: the inclusion (in the synthetic component) of such proteins as cytokines (120), or alternatively, inducers of maturation of DCs (121), the addition of adjuvants (122), and the use of microspheres (123).

### 3.7 Animals

**Figure 7:**
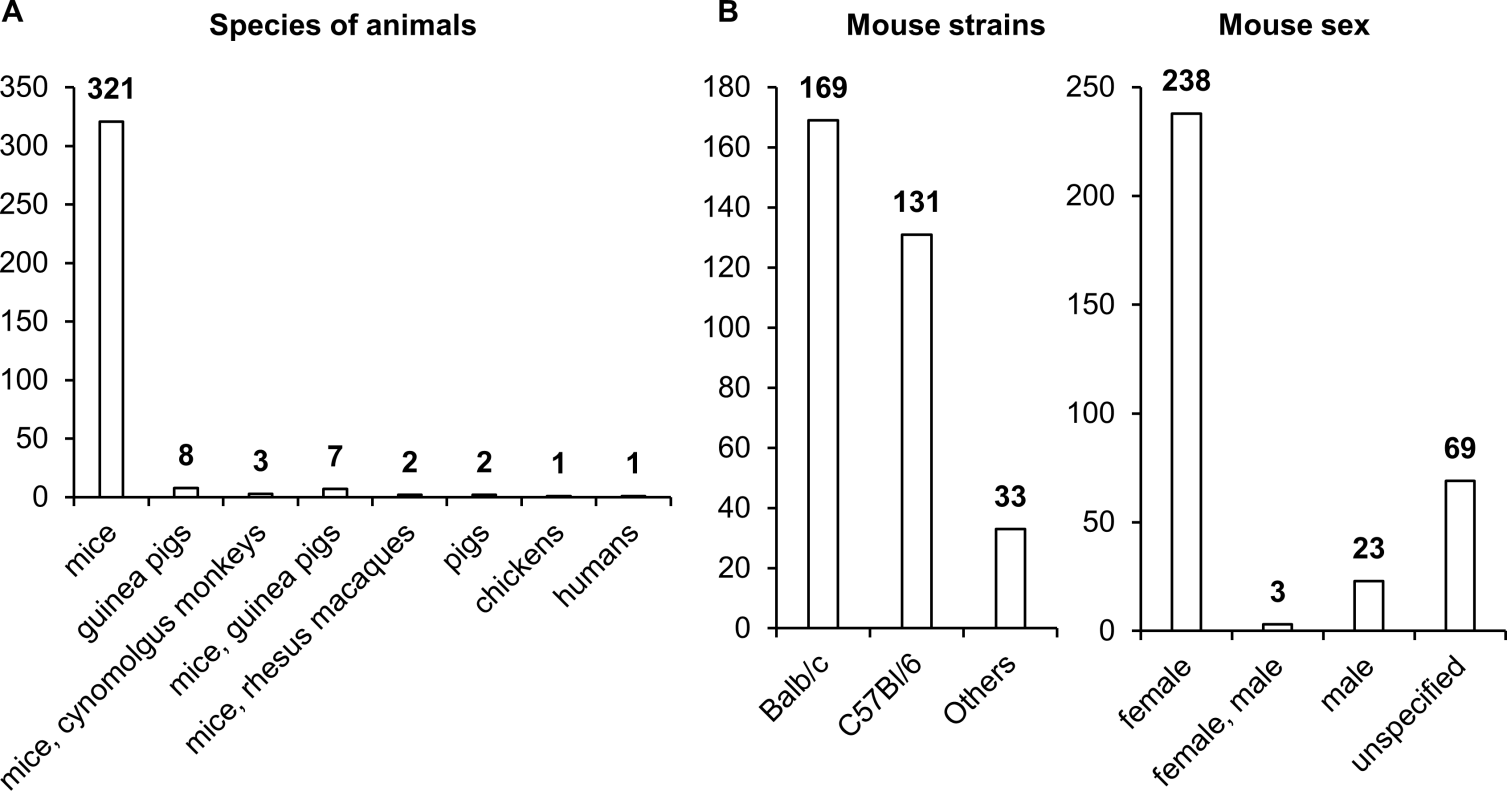
Vaccinated animals. **(A)** Species of vaccinated animals. **(B)** Strains of vaccinated animals. **(C)** Sex of vaccinated animals. The numbers indicate the number of studies in which different species and strains of animals have been used.

Choosing a relevant biological model is always a bottleneck in pharmaceutical research and development. This problem is common in most of nosological entities. Most studies assessing the effectiveness of NA TB vaccines have been performed on female mice, with the BALB/c strain being used most often and C57BL/6 slightly less frequently (**Figure 7**). Other animal species, such as guinea pigs, pigs, chickens, and nonhuman primates, have been used much less frequently in research. Only in 12 studies did researchers assess the effectiveness of vaccines on two animal species.

It is worth noting that differences in the levels of sex steroid hormones affect the functioning of immune cells, leading to variations in immune response activity (124). Specifically, clinical studies indicate that women exhibit higher levels of antibody production and a more pronounced cellular response following vaccination, but they also experience more frequent and severe side effects (124, 125).

The studies on NA-based vaccines included in the analysis were conducted on adult animals, making it difficult to assess their efficacy in offspring and to compare it with the efficacy of the BCG vaccine. For example, it has been shown that approved mRNA vaccines against SARS-CoV-2 demonstrate high efficacy in children, but with lower doses: the COVID-19 vaccine “Comirnaty” by Pfizer/BioNTech for children aged 6 months to 4 years has a dose of 3 µg, and for children aged 5 to 11 years, a dose of 10 µg; the COVID-19 vaccine “Spikevax” by Moderna for children aged 6 months to 4 years has a dose of 2.5 µg, and for children aged 5 to 11 years, a dose of 25 µg (126). Therefore, a more comprehensive assessment of vaccine efficacy in animals of both sexes and different ages is necessary to evaluate the effectiveness of these vaccines.

It should be noted that a biological model for testing TB vaccines should be justified from the point of view of cost-effectiveness (CE) and biological safety (BS) in addition to evidence (E). Although nonhuman primates most closely mimic a human immune response and susceptibility to TB, smaller animal models such as mice, rats, guinea pigs, and rabbits are often better for investigating narrower aspects of the immune response to mycobacteria, e.g., granuloma formation, susceptibility to different strains, or preclinical reactions to the vaccine. In particular, mice are more often employed for evaluating acute toxicity and biodistribution, nonhuman primates for assessing chronic toxicity, guinea pigs for evaluating skin allergic reactions, and rabbits for estimating skin irritation (127). Guinea pigs have high susceptibility to *Mtb* infection, and when infected, they develop classic granulomas similar in structure to human ones.

### 3.8 Comparisons of efficacy between NA vaccines and BCG

At the next stage, out of 345 studies, we selected 17 that assessed protective efficacy of vaccines and involved a BCG vaccine as a control. Out of the selected 17 studies, in six papers, a humoral immune response was assessed in comparison with BCG, and in five of them, an IgG titer after the administration of NA vaccines was higher than after the administration of BCG, and this increase varied from 1.2-fold (128) to 33-fold (129) (**Table 1**). Although injection of NA TB vaccines can induce a humoral response in most cases, induction of IgG production has not always correlated with a highly effective cellular response and with a reduction in the bacterial load of *Mtb* in the lungs and spleen (128, 130, 131). The absence of a direct relation between the effectiveness of a vaccine and a pronounced humoral response has also been documented in comparisons of RNA and DNA vaccines: despite the absence of significant differences in an IgG titer, there have been pronounced differences in the protective effect (130). DNA vaccine pDNA Rv2660c, despite a high level of IgG, has been worse than other vaccines at reducing the number of colony-forming units (CFUs) in lungs (131). Similar effects have been observed with DNA vaccine pDNA Hsp65, which has manifested low protective efficacy as compared to DNA vaccines that have yielded lower IgG titers (128).

**Table 1:** Comparisons of humoral responses after immunization with NA TB vaccines or BCG.

Vaccines		IgG titer			Reference
Gene sequences	Nucleic acid	Negative control	Positive control (BCG)	Experimental	
**MPT83**	DNA	IgG (OD_450_): 0.25	IgG (OD_450_): 0.5	**IgG (OD_450_): 2.75**	(130)
**MPT83**	RNA			**IgG (OD_450_): 2.75**	
Rv3615c	DNA	IgG (OD_450_): 0.3	IgG (OD_450_): 3	IgG (OD_450_): 2.32	(131)
Mtb10.4				IgG (OD_450_): 0.65	
Rv2660c				IgG (OD_450_): 1.79	
Rv3615c, Mtb10.4, Rv2660c				IgG (OD_450_): 2.84	
5 T-cell epitopes from ESAT6, Ag85B, MTB10.4, PPE25, PE19	DNA	IgG (OD_450_): 0.1 IgG2a/IgG1: 0	IgG (OD_450_): 1.4 IgG2a/IgG1: 1.2	IgG (OD_450_): 0.1 IgG2a/IgG1: 0	(128)
Hsp65				IgG (OD_450_): 1.95 IgG2a/IgG1: 0.9	
Hsp65 + 5 epitopes				IgG (OD_450_): 1.6 IgG2a/IgG1: 1	
**5 epitopes into Hsp65 backbone**				**IgG (OD_450_): 1.7** **IgG2a/IgG1: 1.4**	
**ESAT6, CFP10**	DNA	IgG (OD_490_): 0.04	IgG (OD_490_): 0.05	**IgG (OD_490_): 0.25**	(128)
**ESAT6, CFP10**	BCG prime + DNA boost			**IgG (OD_490_): 0.43**	
**CFP21, MTP64**	DNA	IgG (OD_490_): 0.08	IgG (OD_490_): 0	**IgG (OD_490_): 0.24**	
**CFP21, MTP64**	BCG prime + DNA boost			**IgG (OD_490_): 0.19**	
**Ag85B, ESAT6, HspX**	DNA	IgG (OD_450_): 0.18 IgG2a/IgG1: 0.9	IgG (OD_450_): 0.47 IgG2a/IgG1: 1.4	**IgG (OD_450_): 1.05** **IgG2a/IgG1: 4**	(134)
**HspX**				**IgG (OD_450_): 0.7** **IgG2a/IgG1: 2.2**	
**3 epitopes from ESAT6**	DNA	IgG (OD_570_): 0.05	IgG (OD_570_): 0.05	**IgG (OD_570_): 0.5**	(129)
**3 epitopes from ESAT6**	DNA prime + ESAT6 boost			**IgG (OD_570_): 0.72**	
**3 epitopes from ESAT6 + FL**	DNA			**IgG (OD_570_): 0.75**	
**3 epitopes from ESAT6 + FL**	DNA prime + ESAT6 boost			**IgG (OD_570_): 1.35**	
**ESAT6**	DNA			**IgG (OD_570_): 0.7**	
**ESAT6**	DNA prime + ESAT6 boost			**IgG (OD_570_): 0.85**	
**ESAT6 + FL**	DNA			**IgG (OD_570_): 0.8**	
**ESAT6 + FL**	DNA prime + ESAT6 boost			**IgG (OD_570_): 1.65**	

Vaccines that showed better effectiveness as compared to BCG are highlighted in bold.

In addition to antibody levels, two of the six studies assessed the IgG2a/IgG1 ratio. High IgG2a/IgG1 ratios indicate a tendency to activate a Th1-type immune response and in some cases help to suppress the development of the disease; such ratios also have not allowed to determine with certainty whether a vaccine will lead to the synthesis of proinflammatory cytokines and to an enhanced protective effect. Of note, the level of IgG has also been influenced—aside from the antigen being administered—by the method of its delivery, the vaccination regimen, and the applied adjuvants (93, 128, 129, 132). Vaccines containing different types of NAs have not had a substantial effect on IgG levels: both RNA vaccines and DNA vaccines have elicited similar levels of antibody production (130). The antigens within a DNA vaccine that have caused the greatest humoral response are Ag85A, Ag85B, and Hsp65 (113, 128, 133, 134).

For instance, in all the six analyzed studies where humoral immunity was assessed, DNA vaccines in the form of plasmids have been used, and in one study, an RNA vaccine was employed. Furthermore, in all the studies, a homobooster vaccination regimen has been used, and only in two articles have heterobooster vaccination regimens been tested: a BCG prime + a plasmid DNA boost and a plasmid DNA prime + a peptide boost. In one work, a gene coding for the FL adjuvant was utilized, and in three studies of the six, the ESAT6 antigen has been used.

A cellular immune response has been evaluated with the help of the following parameters: ELISA measurement of the level of cytokine production by splenocytes in response to an antigen (i), quantification of cytokine RNA in lymphocytes by reverse-transcription quantitative PCR (ii), an assay of CTL activity by an analysis of CTLs (iii), determination of the frequency of IFN-γ–secreting cells by the ELISPOT method (iv), evaluation of frequencies of cytokine-producing CD4^+^ or CD8^+^ T cells by flow cytometry (v), and quantitation of splenocyte proliferation by the MTT assay (vi) (**Table 2**).

**Table 2:** Comparisons of cellular responses after immunization with NA TB vaccines or BCG.

Vaccines		Cytokine response			Reference
Gene sequences	Nucleic acid	Negative control	Positive control (BCG)	Gene sequences	
**MPT83**	DNA	IFN-γ (pg/ml): 100	IFN-γ (pg/ml): 100	**IFN-γ (pg/ml): 1500**	(130)
**MPT83**	RNA			**IFN-γ (pg/ml): 900**	
Rv3615c	DNA	IFN-γ (pg/ml): 250 TNF-a (pg/ml): 80 IL-2 (pg/ml): 60 IL-4 (pg/ml): 20 IL-10 (pg/ml): 1050	IFN-γ (pg/ml): 1900 TNF-a (pg/ml): 800 IL-2 (pg/ml): 220 IL-4 (pg/ml): 20 IL-10 (pg/ml): 750	IFN-γ (pg/ml): 1050 TNF-a (pg/ml): 400 IL-2 (pg/ml): 120 IL-4 (pg/ml): 20 IL-10 (pg/ml): 1100	(131)
Mtb10.4				IFN-γ (pg/ml): 1000 TNF-a (pg/ml): 380 IL-2 (pg/ml): 90 IL-4 (pg/ml): 20 IL-10 (pg/ml): 750	
Rv2660c				IFN-γ (pg/ml): 800 TNF-a (pg/ml): 360 IL-2 (pg/ml): 110 IL-4 (pg/ml): 20 IL-10 (pg/ml): 850	
Rv3615c, Mtb10.4, Rv2660c				IFN-γ (pg/ml): 1800 TNF-a (pg/ml): 760 IL-2 (pg/ml): 195 IL-4 (pg/ml): 20 IL-10 (pg/ml): 700	
**ESAT6, CFP10**	DNA	IFN-γ (pg/ml): 90	IFN-γ (pg/ml): 125	**IFN-γ (pg/ml): 450**	(128)
**ESAT6, CFP10**	BCG prime, DNA boost			**IFN-γ (pg/ml): 260**	
**CFP21, MTP64**	DNA	IFN-γ (pg/ml): 120	IFN-γ (pg/ml): 120	**IFN-γ (pg/ml): 320**	
**CFP21, MTP64**	BCG prime, pDNA boost			**IFN-γ (pg/ml): 470**	
ESAT6, Ag85B	DNA	―	IFN-γ: 7.3↑ (as compared with negative control) IL-4: 3.4↑	IFN-γ: 2.5↑ IL-4: 2.8↑	(146)
**Ag85B, ESAT6, HspX**	DNA	IFN-γ (pg/ml): 50 IL-2 (pg/ml): 16	IFN-γ (pg/ml): 510 IL-2 (pg/ml): 37	**IFN-γ (pg/ml): 980** **IL-2 (pg/ml): 85**	(134)
**HspX**				**IFN-γ (pg/ml): 570** **IL-2 (pg/ml): 40**	
MPT-32	DNA	IFN-γ (spots/10^6^ cells): 80	IFN-γ (spots/10^6^ cells): 1300	IFN-γ (spots/10^6^ cells): 290	(147)
MPT-63		IFN-γ (spots/10^6^ cells): 100		IFN-γ (spots/10^6^ cells): 260	
MPT-83		IFN-γ (spots/10^6^ cells): 0		IFN-γ (spots/10^6^ cells): 60	
α-crystallin		IFN-γ (spots/10^6^ cells): 0		IFN-γ (spots/10^6^ cells): 0	
Pst-S		IFN-γ (spots/10^6^ cells): 20		IFN-γ (spots/10^6^ cells): 65	
3 epitopes from ESAT6	DNA	IFN-γ (pg/ml): 0 IL-12 (pg/ml): 0 IL-4 (pg/ml): 28 IL-10 (pg/ml): 150	IFN-γ (pg/ml): 390 IL-12 (pg/ml): 280 IL-4 (pg/ml): 75 IL-10 (pg/ml): 170	IFN-γ (pg/ml): 260 IL-12 (pg/ml): 230 IL-4 (pg/ml): 22 IL-10 (pg/ml): 90	(129)
3 epitopes from ESAT6	DNA prime + ESAT6 boost			IFN-γ (pg/ml): 340 IL-12 (pg/ml): 280 IL-4 (pg/ml): 20 IL-10 (pg/ml): 85	
3 epitopes from ESAT6 + FL	DNA			IFN-γ (pg/ml): 310 IL-12 (pg/ml): 290 IL-4 (pg/ml): 20 IL-10 (pg/ml): 70	
**3 epitopes from ESAT6 + FL**	DNA prime + ESAT6 boost			**IFN-γ (pg/ml): 420** **IL-12 (pg/ml): 370** **IL-4 (pg/ml): 18** **IL-10 (pg/ml): 72**	
ESAT6	DNA			IFN-γ (pg/ml): 260 IL-12 (pg/ml): 240 IL-4 (pg/ml): 25 IL-10 (pg/ml): 100	
ESAT6	DNA prime + ESAT6 boost			IFN-γ (pg/ml): 340 IL-12 (pg/ml): 310 IL-4 (pg/ml): 19 IL-10 (pg/ml): 80	
ESAT6 + FL	DNA			IFN-γ (pg/ml): 310 IL-12 (pg/ml): 320 IL-4 (pg/ml): 20 IL-10 (pg/ml): 75	
**ESAT6 + FL**	DNA prime + ESAT6 boost			**IFN-γ (pg/ml): 450** **IL-12 (pg/ml): 400** **IL-4 (pg/ml): 16** **IL-10 (pg/ml): 60**	
3 epitopes from ESAT6	DNA in nano-chitozan particles	IFN-γ (pg/ml): 10 IL-12 (pg/ml): 20 IL-4 (pg/ml): 27 IL-10 (pg/ml): 145	IFN-γ (pg/ml): 370 IL-12 (pg/ml): 250 IL-4 (pg/ml): 75 IL-10 (pg/ml): 195	IFN-γ (pg/ml): 340 IL-12 (pg/ml): 260 IL-4 (pg/ml): 19 IL-10 (pg/ml): 55	(135)
**3 epitopes from ESAT6 + FL**				**IFN-γ (pg/ml): 420** **IL-12 (pg/ml): 340** **IL-4 (pg/ml): 15** **IL-10 (pg/ml): 45**	
**3 epitopes from ESAT6 + FL**	DNA			**IFN-γ (pg/ml): 330** **IL-12 (pg/ml): 280** **IL-4 (pg/ml): 18** **IL-10 (pg/ml): 60**	
**ESAT6**	DNA in nano-chitozan particles			**IFN-γ (pg/ml): 350** **IL-12 (pg/ml): 300** **IL-4 (pg/ml): 17** **IL-10 (pg/ml): 80**	
**ESAT6 + FL**				**IFN-γ (pg/ml): 440** **IL-12 (pg/ml): 350** **IL-4 (pg/ml): 15** **IL-10 (pg/ml): 50**	
**Ag85B**	DNA	IFN-γ (pg/ml): 100	IFN-γ (pg/ml): 2400	**IFN-γ (pg/ml): 6900**	(137)
MPT64				IFN-γ (pg/ml): 1000	
MPT63				IFN-γ (pg/ml): 1200	
ESAT6				IFN-γ (pg/ml): 800	
**Hsp65**	DNA	IFN-γ (spots/10^6^ cells): 220 IL-4 (spots/10^6^ cells): 50	IFN-γ (spots/10^6^ cells): 190 IL-4 (spots/10^6^ cells): 60	**IFN-γ (spots/10^6^ cells): 350** **IL-4 (spots/10^6^ cells): 80**	(93)
**Hsp65**	DNA into microspheres			**IFN-γ (spots/10^6^ cells): 305** **IL-4 (spots/10^6^ cells): 60**	
**Hsp65**	BCG prime, pDNA boost			**IFN-γ (spots/10^6^ cells): 440** **IL-4 (spots/10^6^ cells): 50**	
**Hsp65**	DNA into liposomes			**IFN-γ (spots/10^6^ cells): 390** **IL-4 (spots/10^6^ cells): 10**	

Vaccines that showed better effectiveness as compared to BCG are highlighted in bold.

Of the 17 chosen papers, 10 studies have compared cytokine expression or assessed the frequency of IFN-γ–secreting T cells after NA vaccination and after BCG vaccination; in seven of them, these parameters after administration of an NA have been higher than after the administration of BCG. In almost all studies, the ability of splenocytes to secrete IFN-γ in response to stimulation with a specific antigen has been evaluated to assess the magnitude of a cellular response. IFN-γ is a major cytokine in the anti-TB response because its overexpression positively correlates with a decline of bacterial load. The increase in the IFN-γ level as compared to BCG administration has ranged from 1.1- to 15-fold.

Another key cytokine in the fight against *Mtb* is IL-2. In most cases, greater synthesis of IL-2 by splenocytes in response to inactivated H37Rv and antigens Ag85 and HspX has correlated with the effectiveness of a TB vaccine (131, 133, 134); however, a comparison with BCG has been made in only two studies, and only in ref (134). was IL-2 upregulation by 2.3-fold demonstrated. IL-4 and IL-10—anti-inflammatory cytokines—weaken an anti-TB response, thereby leading to higher bacterial loads (93, 129, 131, 133, 135). In three out of the 10 papers, levels of IL-4 and IL-10 have been measured in comparison with BCG administration, and in two papers, they were 4.4 and 2.6 times lower (129) and 5 and 4.3 times lower (135), respectively, in NA vaccine groups as compared to BCG groups. In the same publications, IL-12 was quantified, and fms-like tyrosine kinase ligand 3 (FL) served as an encoded adjuvant. When vaccines with FL were administered, during activation of splenocytes by the ESAT6 antigen, the highest levels of IFN-γ and IL-12 and diminished levels of IL-4 and IL-10 were observed (129, 135). It should also be pointed out that it was FL in combination with antigen ESAT6—as well as nanochitosan as a delivery system—that led to the greatest protective effect, by reducing the bacterial load in the lungs by 2.5-fold and in the spleen by 1.8-fold more strongly than BCG did.

Application of IL-12 as an encoded DNA vaccine adjuvant has reduced CFU counts in the lungs and spleen to the same extent as application of IL-23, whereas the use of IL-27 has not significantly reduced CFU counts either in the lungs or in the spleen (132). High levels of TNF, along with IFN-γ, are an important modulator of the immune response against *Mtb*; similarly to IFN-γ, IL-2 and IL-12 have decreased CFU counts in the lungs and spleen of mice. Notably, the administration of different types of NAs in vaccines has significantly affected the amount of IFN-γ produced by splenocytes after activation by MPT83: DNA-based vaccines are 1.6 times more effective than RNA-based vaccines (130). For instance, eight of the 10 articles have compared cytokine levels after administration of NA vaccines and after BCG administration, and in five studies, cytokine expression after the injection of plasmid DNA/RNA has exceeded cytokine expression seen after BCG vaccination. In all the eight papers, the level of IFN-γ has been measured, whereas TNF was quantified only in one paper, IL-12 and IL-2 in two papers, IL-10 in three papers, and IL-4 in four articles.

In some studies, cellular responses to NA vaccines have been examined in more detail. In this way, it has been demonstrated that the administration of a DNA vaccine encoding three antigens (Rv3615c, Mtb10.4, and Rv2660c) causes the proliferation of T cells in the spleen in response to inactivated *Mtb*, and that CD4^+^ T cells proliferate 2.5 times more efficiently than CD8^+^ T cells do. In the same work, cytotoxic activity was assessed, and the experimental vaccine manifested higher effectiveness than that of BCG. Moreover, DNA immunization with a plasmid carrying all three antigens more strongly increased the numbers of multifunctional CD4^+^ and CD8^+^ T cells producing IFN-γ, IL-2, and TNF simultaneously as compared to immunization with either BCG or DNA vaccines encoding only one of the antigens (131). In another work, dealing with DNA vaccines carrying the Hsp65 sequence and epitopes of ESAT6, Ag85B, MTB10.4, PPE25, and PE19 as protective agents, the most effective vaccine type caused a significant increase in the proportion (%) of multifunctional CD4^+^ T cells producing IFN-γ, IL-2, and TNF and monofunctional CD8^+^ T cells producing granzyme B, IFN-γ, TNF, and IL-2 (128). The use of the FL adjuvant in combination with full-length ESAT6 has accelerated T-cell proliferation by 1.5-fold as compared to BCG administration, and the use of a peptide boost has increased this difference to 1.8. Additionally, the boost increased the number of IFN-γ^+^ T cells compared to BCG in groups pIRES-EPS-FL and pIRES-ESAT6-FL; cytolytic activity was significantly higher in DNA groups and was further enhanced by the peptide boost, whereas with BCG vaccination, the percentage of lysis was quite low (129). Among the 10 articles that have compared the activity of a T-cell response to NA vaccines with that of BCG, in six studies, the number of IFN-γ–secreting cells has been assessed, and in four studies, this parameter has been higher than that with BCG vaccination. Out of the studies involving a comparison with BCG, CTL activity has been evaluated in four studies, splenocyte proliferation in three studies, and frequencies of cytokine-producing CD4^+^ and CD8^+^ T cells in one study.

The protective effect of vaccines in terms of bacterial load in the lungs and spleen has been estimated by means of CFUs of *Mtb* after infection. In all the 17 papers, the CFUs have been determined in the lungs, but only 10 papers have assessed the bacterial load in the spleen (**Tables 3**, **4**). Besides, in one work (133), in addition to CFUs, mortality rates in groups and changes in the weight of animals after the infection were assessed. Only four studies have shown that NA vaccines result in a greater reduction in bacterial load in the lungs as compared with BCG. Thus, in most studies, vaccination with BCG has been more effective at protecting from TB.

**Table 3:** A comparison of *Mtb* CFUs in lungs after immunization with NA TB vaccines or BCG.

Vaccines		CFU *Mtb*			Reference
Gene sequences	Nucleic acid	Negative control, log_10_(CFU)	Positive control (BCG), log_10_(CFU)	Experimental, log_10_(CFU)	
MPT83	DNA	Short-term^*^: 10^5.6 Long-term^**^: 10^4.5	Short-term^*^: 10^3.9 Long-term^**^: 10^3.9	Short-term^*^: 10^4.9 Long-term^**^: 10^3.9	(130)
MPT83	RNA			Short-term^*^: 10^4.5 Long-term^**^: 10^4.6	
1818PE_PRS	DNA	10^6.4	10^5.4	10^6.3	(136)
1818PE				10^5.9	
Ag85A	DNA	10^4.75	10^4	10^4.2	(133)
Ag85B		10^5.2	10^4.3	10^4.5	
Ag85C		10^5.2	10^4.3	10^5.1	
Ag85A	DNA	10^5.45	10^3.8	10^5.5	(133)
Ag85A + csp(wt)				10^4.9	
Ag85A + csp(mut)				10^5.4	
Rv3615c	DNA	10^6.3	10^3.4	10^5	(131)
Mtb10.4				10^5.2	
Rv2660c				10^5.4	
Rv3615c, Mtb10.4, Rv2660c				10^3.7	
5 T-cell epitopes from ESAT6, Ag85B, MTB10.4, PPE25, PE19	DNA	10^6.5	10^4.2	10^6.3	(128)
Hsp65				10^5.9	
Hsp65 + 5 epitopes				10^5.8	
5 epitopes into Hsp65 backbone				10^5.4	
ESAT6, CFP10	DNA	10^6.3	10^5.2	10^5.7	(128)
**ESAT6, CFP10**	BCG prime, DNA boost			**10^4.8**	
CFP21, MTP64	DNA			10^5.5	
**CFP21, MTP64**	BCG prime, DNA boost			**10^4.6**	
ESAT6, Ag85B	DNA	―	10^1↓ (as compared to negative control)	10^0.7↓	(146)
Ag85B, ESAT6, HspX	DNA	Preventive: 10^5.4 Therapeutic: 10^5.4	Preventive: 10^4.4	Preventive: 10^4.7 Therapeutic: 10^4.9	(134)
HspX				Preventive: 10^4.9 Therapeutic: 10^5.1	
ESAT6, KatG, MPT63, MPT64	DNA	―	―	85% of BCG effectiveness	(147)
3 epitopes from ESAT6	DNA	10^5.5	10^3.6	10^4.3	(129)
3 epitopes from ESAT6	DNA prime + ESAT6 boost			10^4	
3 epitopes from ESAT6 + FL	DNA			10^4.22	
**3 epitopes from ESAT6 + FL**	DNA prime + ESAT6 boost			**10^3.6**	
ESAT6	DNA			10^4.2	
ESAT6	DNA prime + ESAT6 boost			10^3.8	
ESAT6 + FL	DNA			10^4	
**ESAT6 + FL**	DNA prime + ESAT6 boost			**10^3.55**	
**3 epitopes from ESAT6**	DNA in nano-chitozan particles	10^4.5	10^4	**10^4**	(135)
**3 epitopes from ESAT6 + FL**				**10^3.75**	
**3 epitopes from ESAT6 + FL**	DNA			**10^3.8**	
**ESAT6**	DNA in nano-chitozan particles			**10^3.95**	
**ESAT6 + FL**				**10^3.6**	
Ag85A	DNA	Short-term^*^: 10^5.27 Long-term^***^: 10^6.6	Short-term^*^: 10^4.25 Long-term^***^: ↓>1 log10	Short-term^*^: 10^4.5 Long-term^***^: 10^6	(138)
Ag85A	DNA	4 weeks after challenge: 10^5.43 8 weeks after challenge: 10^5.49	4 weeks after challenge: 10^3.64 8 weeks after challenge: 10^4.03	4 weeks after challenge: 10^4.24 8 weeks after challenge: 10^4.77	(113)
**Ag85B, MPT64, MPT63, ESAT6**	DNA	**10^9.9**	**10^7.3**	**10^7.15**	(137)
Hsp65	DNA	10^6	10^3	10^4.75	(93)
Hsp65	DNA into microspheres			10^4.7	
Hsp65	BCG prime, pDNA boost			10^4.8	
Hsp65	DNA into liposomes			10^4.7	
Ag85B	Combination of DNA	10^6.4	10^5.3	10^6	(132)
Ag85B + IL-12				10^5.75	
Ag85B + IL-23				10^5.78	
Ag85B + IL-27				10^5.9	

^*^infection was implemented 4 weeks after the last vaccine injection; **infection was implemented 6 months after the last vaccine injection; ***infection was implemented at 12 weeks after the last vaccine injection. Vaccines that showed better effectiveness than BCG did are highlighted in bold.

**Table 4:** A comparison of *Mtb* CFUs in the spleen after immunization with NA TB vaccines or BCG.

Vaccines		CFU *Mtb*			Reference
Gene sequences	Nucleic acid	Negative control, log_10_(CFU)	Positive control (BCG), log_10_(CFU)	Experimental, log_10_(CFU)	
1818PE_PRS	DNA	10^5.4	10^4	10^5	(136)
1818PE				10^4.6	
Rv3615c	DNA	10^5.2	10^2.7	10^3.8	(131)
Mtb10.4				10^4.4	
Rv2660c				10^5	
Rv3615c, Mtb10.4, Rv2660c				10^2.9	
5 T-cell epitopes from ESAT6, Ag85B, MTB10.4, PPE25, PE19	DNA	10^5.4	10^3.5	10^5.3	(128)
Hsp65				10^4.8	
Hsp65 + 5 epitopes				10^4.7	
5 epitopes into Hsp65 backbone				10^4.6	
ESAT6, CFP10	DNA	10^5.7	10^4.8	10^5.2	(128)
ESAT6, CFP10	BCG prime, pDNA boost			10^4.2	
CFP21, MTP64	DNA			10^5.2	
CFP21, MTP64	BCG prime, pDNA boost			10^4.2	
ESAT6, Ag85B	DNA	―	10^1↓ (as compared to negative control)	10^0.6↓	(146)
Ag85B, ESAT6, HspX	DNA	Preventive: 10^5.5 Therapeutic: 10^5.4	Preventive: 10^4.9	Preventive: 10^5 Therapeutic: 10^5.1	(134)
HspX				Preventive:10^5.1 Therapeutic: 10^4.9	
3 epitopes from ESAT6	DNA	10^5.5	10^3.55	10^4.2	(129)
3 epitopes from ESAT6	DNA prime + ESAT6 boost			10^4.1	
3 epitopes from ESAT6 + FL	DNA			10^3.95	
3 epitopes from ESAT6 + FL	DNA prime + ESAT6 boost			3.6	
ESAT6	DNA			10^4.1	
ESAT6	DNA prime + ESAT6 boost			10^4	
ESAT6 + FL	DNA			10^3.9	
**ESAT6 + FL**	DNA prime + ESAT6 boost			**10^3.45**	
3 epitopes from ESAT6	DNA in nano-chitozan particles	10^4.4	10^3.8	10^3.95	(135)
**3 epitopes from ESAT6 + FL**				**10^3.7**	
**3 epitopes from ESAT6 + FL**	DNA			**10^3.8**	
**ESAT6**	DNA in nano-chitozan particles			**10^3.75**	
**ESAT6 + FL**				**10^3.55**	
Ag85A	DNA	Long-term^***^: 10^5.6	Long-term^***^: ↓>1 log10	Long-term^***^: 10^4.45	(138)
Ag85B	Combination of pDNA	10^5.3	10^4.3	10^4.65	(132)
Ag85B + IL-12				10^4.67	
Ag85B + IL-23				10^4.66	
Ag85B + IL-27				10^5.1	

^***^infection was implemented 12 weeks after the last vaccine injection. Vaccines that showed better effectiveness as compared to BCG are highlighted in bold.

Vaccination with DNA 1818PE clearly indicates that antigens that do not cause a humoral response—but cause a cellular one—are more effective at protecting against TB in comparison to an antigen that evokes a humoral but not a cellular response (136). In the lungs and spleen, the following vaccine types have afforded protection from *Mtb* at a level equal to or even better than BCG-mediated protection: BCG + ESAT6-CFP10 DNA, BCG + CFP21-MTP64 DNA (128); pIRES-EPS-FL DNA + ESAT6 peptide boost, pIRES-ESAT6-FL DNA + ESAT6 peptide boost (129), nano-ESAT6/3e DNA, nano-ESAT6/3e-FL DNA, ESAT6/3e-FL DNA, nano-ESAT6 DNA, nano-ESAT6-FL DNA (135); and Ag85B DNA + MPT64 DNA + MPT63 DNA + ESAT6 DNA (137). The most commonly used antigens have been Ag85B and ESAT6, with ESAT6 giving the best protection. It is also evident that BCG prime has a beneficial effect on CFU counts, by reducing them 10^0.9^-fold as compared to the administration of DNA alone and by 10^0.4^–10^0.6^-fold as compared to BCG, and the use of a peptide boost reduces the number of CFUs by 10^0.45^-fold as compared to homobooster injection of a DNA vaccine. The addition of adjuvants (FL, IL-12, or IL-23) diminishes CFU counts by 10^0.1^–10^0.4^-fold.

Especially interesting is a study comparing the effectiveness of DNA and RNA vaccines (130). To evaluate a short-term protective effect, mice were infected at 4 weeks—and for a long-term assessment, at 6 months—after the last injection of an NA vaccine; CFUs were determined 5 weeks after the infection. In terms of short-term protection, immunization by either a DNA vaccine or RNA vaccine had a moderate protective effect; however, CFU counts with BCG were an order of magnitude lower. In the experiment on long-term protection, the DNA vaccination reduced CFU counts to the BCG level, and the RNA vaccination was ineffective (130). This difference is likely explained by the fact that antigen expression persists for a longer period when a DNA vaccine is administered in comparison with an RNA vaccine. In another work, where researchers evaluated short- and long-term effects after immunization with self-replicating mRNA vaccine SinCP-Ag85A, vaccination gave a strong protective response in the lungs at 4 weeks after infection, but after 6 months, CFU counts in the lungs increased significantly (138).

In addition to preventive vaccination, one of the studies deals with therapeutic administration of a vaccine 4 weeks after infection (134). The therapeutic vaccination diminished bacterial load in the lungs similarly to preventive vaccination; however, in the spleen, with therapeutic vaccination by means of a plasmid DNA vaccine carrying only one antigen, CFU counts were reduced in the same way as with BCG vaccination.

In an article about survival and weight change in infected mice (133), mean survival time of mice receiving a control vector was 95 days. The onset of death in mice vaccinated with DNA was approximately the same among all groups, regardless of the vaccine administered. Mean survival time of mice vaccinated with p-sAg85A–csp(mut) was identical to mean survival time in a negative control group (95 days). Vaccination with p-sAg85A had no effect on survival either (mean survival time = 102 days). By contrast, five out of 10 mice vaccinated with p-sAg85A–csp(wt) lived longer (than did other mice vaccinated with plasmid DNA) and later began to lose weight because the mean survival time of this group was 151.5 days, which is 50 days more than the mean survival time of mice vaccinated with p-sAg85A, and 14 days more than that of mice vaccinated with BCG (mean survival time = 140.3 days).

As mentioned above, in only four of the 17 examined articles, the effectiveness of NA vaccines is superior to that of BCG, which is still the only vaccine against *Mtb*. Even though only a minority of the tested NA vaccines reduce the number of CFUs better than BCG does, these findings provide new insights into immunization against *Mtb*.

The fundamental advantages of NA-based vaccines, classified as third-generation vaccines, include the rapid development process, the ability to flexibly select antigenic compositions, and a relatively inexpensive and scalable production process. NA-based vaccines enable the “fine-tuning” of the optimal balance between humoral and cellular immunity, particularly Th1/Th2 immunity. This adjustment could be effective in the context of tuberculosis, as one of the potential causes of disease progression is the imbalance between Th1/Th2/Th17 cells due to an excessive shift toward the Th2 response, which suppresses the action of Th1 cytokines (139). NA-based vaccines have a broad range of tools for modulating immune responses. In addition to the possible use of classical adjuvants, genetic adjuvants, which have demonstrated their effectiveness (91), can also be employed. Another strategy is the creation of multi-epitope vaccines that encode only specific epitopes of target antigens. *In silico* tools allow for the selection of optimal CTL, HTL, and LBL epitopes, as well as predicting which cytokines will be induced after antigen presentation as part of MHCII and binding with helper T-cell receptors. This approach has proven effective in our recent study on the development of mRNA vaccines against tuberculosis (140).

However, it should be acknowledged that in the context of tuberculosis vaccine development, the use of *in silico* algorithms and artificial intelligence methods, such as deep learning and other forms of machine learning, is still in its early stages (141). *In silico* approaches facilitate the selection of optimal epitopes, the determination of the secondary and tertiary structure of target polypeptides, molecular docking, and even the prediction of potential immune response dynamics and immunity formation. This enables the selection of the optimal vaccination regimen. Nevertheless, the majority of *in silico* studies related to tuberculosis vaccine development have yet to be experimentally validated for their efficacy in animal models (142, 143). It appears that several more years, or even decades, of comprehensive research (combining *in silico* prediction and experimental validation) will be necessary to achieve an effective vaccine, with continuous optimization of algorithms based on the data obtained. Nonetheless, we believe that the prospects for using *in silico* approaches in the development of NA vaccines, particularly mRNA vaccines, are promising. Taking together, with appropriate adjuvants and antigens, with optimization of delivery systems, and after the best immunization regimen is found, NA vaccines will be able to outperform BCG.

## 4 Discussion and conclusion

In our review, we extensively analyzed 345 *in vivo* animal studies that evaluated the efficacy of NA-based vaccines against tuberculosis. These studies evaluated the antigenic sequence, delivery systems, routes of administration, adjuvants, and mode and strategy of administration of the vaccine formulation. In addition, animal models for testing the efficacy of NA-based vaccines were evaluated and a comparison of their efficacy compared to BCG vaccine was provided.

The review design is associated with several strengths. We employed rigorous processes for identifying, selecting, summarizing, and reporting evidence, working from a pre-registered protocol and adhering to state-of-the-art standards for knowledge synthesis. The search strategy was peer-reviewed by an information specialist and run across a comprehensive range of relevant databases and registers, supplemented with manual review of gray literature sources and citation searches. To minimize the risk of selection bias, each record and report was screened independently by two reviewers, with the inter-rater agreement reached through a sufficiently powered piloting procedure. A pre-registered data charting form was enforced to ensure consistent data collection. Reports in languages other than English were not discarded. Raw review data, including over 60 hours of video content, were published to promote process transparency and support the reproducibility of findings.

The conduct of the review was associated with several shortcomings, mostly arising from deviations from the published protocol. We could not access several ProQuest databases, reduced the number of records retrieved from Google Scholar from 1000 to 100, canceled forward citation searches, only searched in English, and provided a limited coverage of local databases, contributing to the risk of bias against gray literature and publications in other languages. Further, we failed to find or access online 10.5% of studies deemed eligible by title and abstract (113 of 1081), with most of them originating from China or published in the previous century. These risks were partially mitigated by the fact that multiple studies included in this review were conducted outside of the Commonwealth or the US, with China emerging as the leading region of affiliation. The last date of search was in September 2022; however, only 13.9% of the included studies were published after 2015 (48 of 345), at a rate of fewer than 10 per year and declining. Our published open data can be used to rerun the searches and update the review findings. Lastly, we refrained from charting in duplicate due to the large number of included reports, which could reduce data accuracy, even though we conducted informal consistency checks.

Although our results are based on a considerable number of experimental studies over the last few decades, there are several limitations to the application of these results.

The first limitation is related to the significant heterogeneity of study designs, vaccine dosages used, and methodologies for assessing the efficacy of vaccine formulations. Conducting comprehensive studies on animals of different species using standardized techniques could greatly facilitate comparison of the efficacy of NA-based vaccine preparations.

The second limitation is related to the small number of available analytical data on the vaccine preparation in most studies. The degree of purity of the preparation (RNA/DNA *E. coli*, endotoxins, etc.), the specificity of the active pharmaceutical substance, and the presence of other impurities may affect its efficacy. The availability of generally accepted standards for characterization of NA-based vaccines for all *in vivo* studies would help to address this issue.

Another limitation to the interpretation of the results is related to the significant progress in molecular biology, biotechnology, nanotechnology, and artificial intelligence over the past two decades. In the context of NA-based vaccine development, this progress relates to the development of efficient delivery systems, the development of highly sensitive systems for *in vivo* imaging of NA-based drug components or their protein products after administration, allowing the biodistribution of drug prototypes to be assessed using specific reporters. In addition, the application of *in silico* methods to assess putative efficacy allows the selection of optimal nucleic acid sequences. Separately, significant progress in the development of mRNA vaccines should be mentioned, including the use of uridine analogs and the development of an efficient delivery system based on lipid nanoparticles. Most of these tools were not used in early studies, and only a fraction of research teams have used them in more recent studies.

Due to this progress, many new vaccine development strategies have become possible. Recent findings about mRNA vaccines against tuberculosis (both those included in this review and those published more recently) and data from clinical trials of the BNT164a1/BNT164b1 vaccines developed by BioNTech are a reason for considerable optimism in this complicated field (144, 145). It is imperative to maintain the momentum gained over the past 2 decades so that *Mtb* — a pathogen that has been with us for 3 million years — is finally doomed to the same fate as smallpox.

Although the development of a TB vaccine has historically faced major obstacles, it is important to recognize the substantial progress that was made recently both in the elucidation of the immunology involved and in empirical research (preclinical and clinical trials) in humans. The immunological understanding of the interaction of *Mtb* with the host has slowly but surely painted the clearer picture that we have today. The classic view of TB — with a focus solely on the adaptive response — has evolved into something much more complex, by integrating the innate, adaptive, and humoral systems.

Active studies on animal models have revealed that an effective vaccine against *Mtb* must meet the following criteria: firstly, the induction of the “correct” ratio of T-cell subsets and the “correct” cytokine spectrum along with a rapid response to the infection combined with the induction of protective long-lasting immunity (immunological memory); secondly, activation of the “correct” effector mechanisms aimed at preventing the infection or eliminating the infectious agent; thirdly, high specificity for the infectious agent to minimize the risk of autoimmunity due to cross-reactive antigens. Furthermore, NA vaccines must express antigens found in all isolates and strains of the pathogen, and these antigens must be immunogenic for all MHC haplotypes of the human population.

## Data Availability

Publicly available datasets were analyzed in this study. This data can be found here: https://osf.io/x95hj/.
