# Supplementary material for: DNA and RNA vaccines against tuberculosis: a scoping review of human and animal studies
Source: Front Immunol. 2024 Oct 3;15:1457327. doi: 10.3389/fimmu.2024.1457327 (PMC11483866; doi:10.3389/fimmu.2024.1457327)
Supplement: Supplementary file 1 [file Table1.docx]

✅ **Supplementary Table 1.** Reporting checklist to assess the state of reporting within preclinical systematic reviews.

| Section | # | Item | Reported on page # |
| --- | --- | --- | --- |
| Title | 1 | Identify the report as systematic review in title | Modified for scoping review |
|  | 2 | Identify that the report contains animal data in title (preclinical, *in vivo* or synonym) | ✅ |
| Intro | 3 | Describe the human condition being modelled (e.g. describe what is already known) | ✅ |
|  | 4 | Describe the biological rationale for testing the intervention (e.g. how would the intervention affect the condition) | ✅ |
|  | 5 | Provide an explicit statement of the question(s) the review addresses (specify the main objectives of the review, ideally in PICO format) | ✅ |
| Methods | 6 | Indicate whether a review protocol was registered *a priori* | ✅ |
|  | a | Where can the protocol be accessed and indicate the name of the protocol registry OR state that it is not available | ✅ |
|  | b | Indicate any deviations from the protocol OR that there were no deviations | ✅ |
|  | 7 | Eligibility criteria: Describe the animal species to be included in the review (e.g. only mice, vertebrates, large animals) | ✅ |
|  | 8 | Eligibility criteria: Describe the animal model to be included in the review (methods of disease induction, age, sex, etc.) | ✅ |
|  | 9 | Eligibility criteria: Describe the intervention/exposure of interest | ✅ |
|  | 10 | Eligibility criteria: Describe the comparators and/or control population | Not applicable |
|  | 11 | Eligibility criteria: Describe the primary outcomes of interest (what is being measured/assessed in primary studies) | ✅ |
|  | 12 | Eligibility criteria: Describe the timing (prevention vs rescue) of intervention, IF applicable | Not applicable |
|  | 13 | Indicate where a full search strategy of all data bases OR representative search strategy can be accessed | ✅ |
|  | 14 | Describe inclusion limits (years conducted, language, AND publication type) | ✅ |
|  | 15 | Describe the study screening/selection process | ✅ |
|  | a | Report the platform used to screen and select studies (Excel, Access, DistillerSR, SyRF) | ✅ |
|  | 16 | State the number of independent screeners | ✅ |
|  | 17 | Describe methods for extracting numerical data from reports (e.g. data in bar graph, or non-text presentation), IF applicable * | ✅ |
|  | a | Report the platform and tools used to extract numerical data (Graph2data, Engauge) | Not applicable |
|  | 18 | Report number of independent reviewers extracting data | ✅ |
|  | 19 | Describe methods and tool used to measure study quality/risk of bias in individual studies (e.g. SYRCLE tool, CAMARADES tool) | ✅ |
|  | 20 | Describe methods to assess construct validity in individual studies | Not applicable |
|  | 21 | Describe methods for assessing publication bias of included studies, IF applicable | Not applicable |
|  | 22 | Describe methods for synthesizing the quantitative effect measures of included studies (e.g. risk ratio, mean difference), IF applicable * | Not applicable |
|  | 23 | Describe methods for any data transformation needed to make extracted data suitable for analysis (e.g. only sample size range), IF applicable * | Not applicable |
|  | 24 | Describe methods for handling shared control groups (common issue in analysis of preclinical studies), IF applicable * | Not applicable |
|  | 25 | Describe methods for assessing heterogeneity between individual studies, IF applicable * | Not applicable |
|  | 26 | Describe methods for handling effect sizes over multiple time points (e.g. used all time points or latest time point), IF applicable * | Not applicable |
|  | 27 | Describe methods for sub-group and sensitivity analysis, IF applicable * | Not applicable |
| Results | 28 | Report the number of included reports (individual references/publication) included in the review | ✅ |
|  | a | Provides a list or table of individual studies with data or references | All bibliographic files are reported in the review repository |
|  | 29 | Report the number of eligible experiments included in the analysis (eligible animal experiments in individual reports) | Not applicable |
|  | 30 | Include a PRISMA flow diagram (or equivalent) of study selection process | ✅ |
|  | 31 | Study characteristics: Report animal species | ✅ |
|  | 32 | Study characteristics: Report animal model details (e.g. method of disease induction, age, sex) | ✅ |
|  | 33 | Study characteristics: Report a measure of the sample size (e.g. total number or mean number of animals) | Not applicable |
|  | 34 | Study characteristics: Report intervention/exposure details (timing, dose) | ✅ |
|  | 35 | Study characteristics: Report study design/intention (pharmakinetic, mechanistic, efficacy) | ✅ |
|  | 36 | Report the risk of bias of the primary studies (individual studies/across outcomes) | Not applicable |
|  | 37 | Report the outcome effects of primary studies (forest plot if applicable), IF applicable * | Not applicable |
|  | 38 | Report the confidence intervals of outcomes for the included studies, IF applicable * | Not applicable |
|  | 39 | Report any measure of heterogeneity between studies, IF applicable * | Not applicable |
|  | 40 | Report the results of sub-group and sensitivity analysis, IF applicable * | Not applicable |
|  | 41 | Report the results of publication bias, OR report that it was not possible/done | Not applicable |
| Discussion | 42 | Discuss the impact of the risk of bias of the primary studies | Not applicable |
|  | 43 | Discuss the limitations (i.e. limitation of primary studies and/or outcomes included) | ✅ |
|  | 44 | Discuss the limitations of the systematic review | ✅ |
| Other | 45 | Include the funding source(s) of the systematic review | ✅ |
|  | 46 | Report any data sharing, OR that there was no data sharing | ✅ |
| * Reporting item is not applicable to systematic reviews that did not perform a quantitative synthesis. For reviews that did not perform a quantitative synthesis, these items receive an NA. | | |  |

*From:* Hunniford VT, Montroy J, Fergusson DA, Avey MT, Wever KE, McCann SK, Foster M, Fox G, Lafreniere M, Ghaly M, Mannell S, Godwinska K, Gentles A, Selim S, MacNeil J, Sikora L, Sena ES, Page MJ, Macleod M, Moher D, Lalu MM. Epidemiology and reporting characteristics of preclinical systematic reviews. PLoS Biol. 2021 May 5;19(5):e3001177. doi: 10.1371/journal.pbio.3001177.
